# Exploring the Impact of Cleavage and Polyadenylation Factors on Pre-mRNA Splicing Across Eukaryotes

**DOI:** 10.1534/g3.117.041483

**Published:** 2017-05-08

**Authors:** Gildas Lepennetier, Francesco Catania

**Affiliations:** Institute for Evolution and Biodiversity, University of Münster, 48149, Germany

**Keywords:** AAUAAA, splicing, polyadenylation, U1 snRNP, transcription, gene

## Abstract

In human, mouse, and *Drosophila*, the spliceosomal complex U1 snRNP (U1) protects transcripts from premature cleavage and polyadenylation at proximal intronic polyadenylation signals (PAS). These U1-mediated effects preserve transcription integrity, and are known as telescripting. The watchtower role of U1 throughout transcription is clear. What is less clear is whether cleavage and polyadenylation factors (CPFs) are simply patrolled or if they might actively antagonize U1 recruitment. In addressing this question, we found that, in the introns of human, mouse, and *Drosophila*, and of 14 other eukaryotes, including multi- and single-celled species, the conserved AATAAA PAS—a major target for CPFs—is selected against. This selective pressure, approximated using DNA strand asymmetry, is detected for peripheral and internal introns alike. Surprisingly, it is more pronounced within—rather than outside—the action range of telescripting, and particularly intense in the vicinity of weak 5′ splice sites. Our study uncovers a novel feature of eukaryotic genes: that the AATAAA PAS is universally counter-selected in spliceosomal introns. This pattern implies that CPFs may attempt to access introns at any time during transcription. However, natural selection operates to minimize this access. By corroborating and extending previous work, our study further indicates that CPF access to intronic PASs might perturb the recruitment of U1 to the adjacent 5′ splice sites. These results open the possibility that CPFs may impact the splicing process across eukaryotes.

There is still much left to be learned about the events that occur throughout the transcription cycle in eukaryotes. The intricacy of the extensive and dynamic network of interactions that link the molecular machineries that contribute to transcript formation (Moore and Proudfoot 2009; Bentley 2014) greatly complicates efforts to close this knowledge gap. While studies of molecular biology and biochemistry have helped unravel a remarkable number of these multifarious interactions (Niwa and Berget 1991; Gunderson *et al.* 1994; Lewis *et al.* 1996; de la Mata *et al.* 2003; Bird *et al.* 2004; Kaida *et al.* 2010; Berg *et al.* 2012; Nojima *et al.* 2015), a thorough elucidation of transcription-coupled events may benefit when we peer beyond the borders of these disciplines. The properties of modern genes, including the mechanism for transcription regulation, result from the interplay of intracellular and selective processes. Thus, considering both the intracellular environment, and the population genetic environment, may provide fresh insights into the processes that unfold during transcription.

Recently, we have put forward two models explaining the widespread properties of eukaryotic genes, wherein conserved interactions between mRNA-associated processes are combined with population genetic processes. In the *intronization* model, exonic sequences may convert into constitutively spliced introns over evolutionary time via a transient phase of alternative splicing (Catania and Lynch 2008; Catania *et al.* 2009; Catania and Schmitz 2015). Today, events of intronization are documented across multiple species (Clarke *et al.* 2013; Zhang and Chasin 2006; Irimia *et al.* 2008; Roy 2009; Zhu *et al.* 2009; Szczesniak *et al.* 2011; Croll and McDonald 2012; Kang *et al.* 2012; Kim and Hahn 2012; Zhu and Niu 2013; Zhan *et al.* 2014). The second model, *U1-dependent definition*, is an extension of the *intronization* model, and accounts for more recent observations of interactions between mRNA-associated processes, such as telescripting (see below) (Catania and Lynch 2013). The *U1-dependent definition* posits that the mechanisms regulating architectural changes, transcriptional activity, and splice site recognition in eukaryotic genes are interdependent. Furthermore, the mechanism for splice site recognition that *U1-dependent definition* supports (*i.e.*, splice sites are recognized across an intron and its next exon) unites the two currently accepted intron definition and exon definition models (Berget 1995).

A prediction of both *intronization* and *U1-dependent definition* is that the interplay between mRNA-associated processes may shape gene structure. In one example, the capacity of the cap-binding complex to enhance the recruitment of the spliceosomal complex U1 locally (Colot *et al.* 1996; Lewis *et al.* 1996) is proposed to facilitate splicing at the gene 5′ end—a condition that could partly explain the frequent 5′ positional bias of introns. We recently tested this prediction employing *Drosophila* as case study. After putting forward a scheme of empirically substantiated expectations about how mRNA-associated processes might sculpt eukaryotic gene structure (based on prior experimental findings), we performed an extensive array of targeted computational analyses to test these expectations. Our observations are consistent with the hypothesis that interacting mRNA-associated processes may impose significant constraints on the exon–intron structure of *Drosophila* (Lepennetier and Catania 2016).

Another central idea put forward in both models is that splicing factors (SFs) and cleavage and polyadenylation factors (CPFs) compete throughout transcription for access to overlapping, or neighboring, signal sequences. To date, this idea remains formally untested.

SFs and CPFs are major players in splicing and in mRNA 3′ end processing, respectively. They are thought to physically interact during transcription (Lutz *et al.* 1996; Awasthi and Alwine 2003; Kyburz *et al.* 2006; Millevoi *et al.* 2006; Lee and Tarn 2014), and to travel associated with the C-terminal domain (CTD) of the RNA polymerase II toward the gene 3′ end (Phatnani and Greenleaf 2006). As transcription progresses, the molar ratio of CTD-bound CPFs to SFs may increase as a result of variation in the phosphorylation status of specific CTD residues (Licatalosi *et al.* 2002; Ahn *et al.* 2004).

Despite their designation, SFs may also affect mRNA 3′ end processing (Gunderson *et al.* 1998; Ashe *et al.* 2000; Vagner *et al.* 2000; Millevoi *et al.* 2006; Wang *et al.* 2008). CPFs, on the other hand, may recognize internal cryptic (rather than only terminal) polyadenylation signals (PAS) (Yao *et al.* 2012; Hoque *et al.* 2013; Shi and Manley 2015) and influence *e.g.*, transcription initiation (Mapendano *et al.* 2010). When CPFs bind to internal PAS, undesired premature cleavage and polyadenylation might be facilitated. Thus, cells have evolved surveillance systems that ensure transcriptome integrity. Studies of human, mouse, and *Drosophila* have demonstrated that the spliceosomal complex U1snRNP (or U1) protects nascent transcripts from premature cleavage and polyadenylation at cryptic intronic PAS in a process dubbed telescripting (Kaida *et al.* 2010). These suppressive effects require that the U1s are bound to authentic or cryptic 5′ splice sites (5′ss). Furthermore, these effects are distance-dependent—they can extend over a median distance of ∼500 nt (in *D. melanogaster*) and ∼1000 nt (in human and mouse) (Berg *et al.* 2012). The mechanism(s) of U1-dependent PAS suppression remains unclear: U1 may directly interact with CPFs (Gunderson *et al.* 1998), and/or it may suppress proximal PAS by disturbing the recruitment of CPFs (Langemeier *et al.* 2013).

The intracellular concentration of U1 can modulate telescripting (Berg *et al.* 2012; Devany *et al.* 2016). For example, Berg *et al.* 2012 demonstrated that a progressive reduction of available U1 produces progressively shorter mRNA isoforms, due to the use of more proximal PAS. Other studies indicate that telescripting could also be modulated by the strength of 5′ss signals, which presumably affects the efficiency of U1 recruitment. In particular, mammalian and plant introns that contain PASs are more likely to undergo alternative polyadenylation when they have a weaker 5′ss (Tian *et al.* 2007; Wu *et al.* 2011). These findings hint at a dynamic competition between SFs and CPFs within introns, which aligns well with predictions of the *intronization* and the *U1-dependent definition* models.

Antagonistic interactions between SFs and CPFs are also consistent with a study wherein a CPF-bound intronic PAS (AATAAA) was found to suppress the inclusion of the upstream exon (Evsyukova *et al.* 2013). The intronic PAS in question is in the vicinity of the 5′ ss signal of exon 6 of the Interleukin 7 receptor gene. A reasonable explanation for this finding is that intron-bound CPFs interfere with the binding of U1 to the adjacent (16 nt upstream) 5′ ss. Intriguingly, the 5′ ss signal in question is weak, and its strengthening fully restores the inclusion of exon 6.

Finally, antagonistic interactions between SFs and CPFs are consistent with observations documented by our laboratory (Lepennetier and Catania 2016). We found that the degree of selective pressure against intronic AATAAA motifs in *Drosophila* is more pronounced within introns that have a relatively weak 5′ss. We have proposed two possible explanations for this observation. First, intronic PAS are suppressed more strongly in the proximity of suboptimal 5′ss signals to compensate for these signals’ inadequate suppression. Second, intron-bound CPFs may perturb the recruitment of U1 at upstream 5′ss.

Here, we used a method based on DNA strand asymmetry (DSA) to gain insights into the relationships between U1 and CPFs during transcription. DSA provides an estimation of the selective pressure for, or against, a specific sequence motif (Zhang *et al.* 2008; Farlow *et al.* 2012; Lepennetier and Catania 2016). We found that the conserved AATAAA PAS is selected against in intronic regions, regardless of the location of the intron along genes, or of the species examined. The counter-selection of AATAAA is especially pronounced in the immediate vicinity of the upstream 5′ss signal, more so when the 5′ss signal is weak. Our observations lend support to the hypothesis that, throughout the transcription cycle, intron-bound CPFs interfere with binding of U1 to adjacent 5′ ss signals.

## Materials and Methods

### Genome sequence and annotations

The names of the 17 eukaryotic species surveyed, and the links we used to retrieve the gene sequences and the relative annotation files, are listed in the (Supplemental Material Table S1 in File S1). We studied ≥2 intron-containing genes, and randomly selected one mRNA isoform for genes with multiple isoforms.

### DNA strand asymmetry of the AATAAA motif

We employed the DNA strand asymmetry of the polyadenylation AATAAA motif (DSA_AATAAA_) as a tool to infer transcription-coupled dynamics of cleavage and polyadenylation factors (CPFs). Under Chargaff’s second parity rule, the asymmetry of nucleotides (or short motifs) between complementary strands of DNA should be null (Mitchell and Bridge 2006). Deviations, *i.e.*, positive (negative) DSA scores, are indicative of selection for (or against) the string under study.

We computed the DSA_AATAAA_ in introns and exons separately, after removing genes nested or hosting another gene from our data. We trimmed intron and exon sequences to minimize the potential impact of the splicing signals (the 5′ and 3′ splice sites and the polypyrimidine tract) on the DSA_AATAAA_ score. Specifically, we did not consider the 3 nt at the ends of exons, 6 nt at the 5′ end, and 40 nt at the 3′ end of introns. In *P*. *tetraurelia*, introns are typically shorter than 40 nt (Aury *et al.* 2006), so we decided to trim only 6 nt at the 5′ end, and 6 at the 3′ end.

### Splice site score

We calculated the strength of the 5′ and 3′ splice site signals using the MaxEntScan scoring method for human and mouse (Yeo and Burge 2004). The MaxEntScan scoring method, retrained using *D*. *melanogaster* splicing signals, was kindly provided by Gene Yeo and Joel McManus (McManus *et al.* 2014).

### Genes with varying expression levels in D. melanogaster

We studied genes that are highly and weakly expressed in *D*. *melanogaster*. Sets were composed of genes with associated expression values that fall in the upper and lower quartile of a distribution of gene expression values generated using ftp://flybase.org/flybase/associated_files/Gelbart.2010.10.13.tar.gz.

### Data availability

All data necessary for confirming the conclusions presented in the article are represented fully within the article. All of the scripts are publicly available through GitHub at https://github.com/GildasLepennetier/GildasSources.

## Results

Recently, we have used a method based on DSA to detect signatures of selection associated with the conserved AATAAA PAS motif in *Drosophila*. The constraints that we uncovered served to generate hypotheses regarding *why* modern eukaryotic genes are shaped the way they are (Lepennetier and Catania 2016). Here, we explore whether the DSA of the AATAAA PAS (or DSA_AATAAA_) within spliceosomal introns may enhance our understanding of *how* SFs and CPFs operate and interact during transcription.

### CPF access to spliceosomal introns is universally disfavored across eukaryotes

We began by computing the DSA_AATAAA_ in the introns of human, mouse, *D*. *melanogaster*, and of 14 additional eukaryotes, including vertebrate and invertebrate animals, the plant *Arabidopsis thaliana*, and the ciliate *Paramecium tetraurelia*. Because CPF access to introns may facilitate unregulated premature cleavage and polyadenylation, we hypothesized that intronic cryptic PASs are generally selected against, unless telescripting adequately suppresses them.

**Figure 1:**
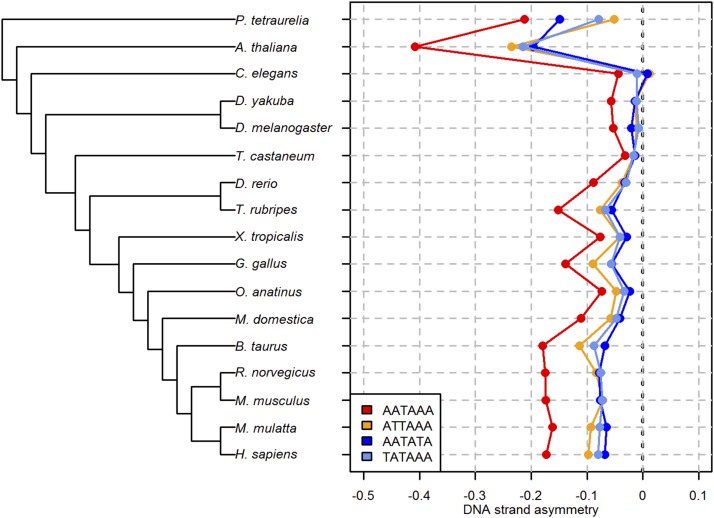
DNA strand asymmetry of polyadenylation(-like) signal motifs in the introns of 17 eukaryotic species. Left panel: approximate phylogenetic relationships spanning ∼2 billion yr between the ciliate *Paramecium tetraurelia* and *Homo sapiens*. Right panel: DNA strand asymmetries of four polyadenylation(-like) signal motifs (AATAAA, ATTAAA, AATATA, and TATAAA).

We found that the AATAAA motif is uniformly counter-selected in the introns of all of the species surveyed (Figure 1). It is also among the six-mers with the most negative DSA in introns (top-5% in 10 species) (Table S2 in File S1). Instead, the AATAAA’s DSA is highly positive in the gene 3′ end of these same species (except *A*. *thaliana* and *T*. *castaneum*, Table S3 in File S1), supporting the idea that this motif operates as canonical PAS in most species. Additionally, the DSA of the AATAAA motif is invariably more negative compared to that of three other hexamers (ATTAAA, AATATA, and TATAAA), which do or may function as PAS across eukaryotes (Beaudoing *et al.* 2000; Retelska *et al.* 2006) (Figure 1). Also, with the exception of *T*. *castaneum*, the DSA_AATAAA_ is more negative than our control motifs (DSA_TAAAAA_ and DSA_AAAAAT_), which were selected on the basis of their identical nucleotide composition to, and partial overlap with, AATAAA, and the fact that they should not operate as PAS (Table S2 in File S1**)**. Altogether, these findings support the hypothesis that the AATAAA PAS in spliceosomal introns is generally unfavorable across eukaryotes.

### Intronic AATAAA PASs are counter-selected within the action range of telescripting

The results illustrated in Figure 1 suggest that the magnitude of the selection pressure against the AATAAA motif in introns may vary considerably across species. Between-species differences in the intracellular, and/or the population-genetic, environment can account for these variations. With regard to the intracellular environment, telescripting could modulate this selection pressure. Specifically, because the suppressive effects of U1s are distance-dependent (at least in human, mouse, and *D*. *melanogaster*; Berg *et al.* 2012), it is possible that intronic AATAAA PASs are more strongly suppressed in species with long introns, such as human and mouse, than they are in species with shorter introns, such as *D*. *melanogaster*. If this were the case, one may expect a more negative DSA_AATAAA_ score in human and mouse compared to *D*. *melanogaster*, as is observed (Figure 1).

To formally test this hypothesis, we examined the DSA_AATAAA_ in intronic regions with increasing distances from the upstream 5′ss. Our expectation is that, for long introns, the first few hundred nucleotides (protected by telescripting) have a DSA_AATAAA_ score of ∼0, whereas a signature of negative selection (*i.e.*, DSA_AATAAA_ < 0) should be detected for the following nucleotides.

**Figure 2:**
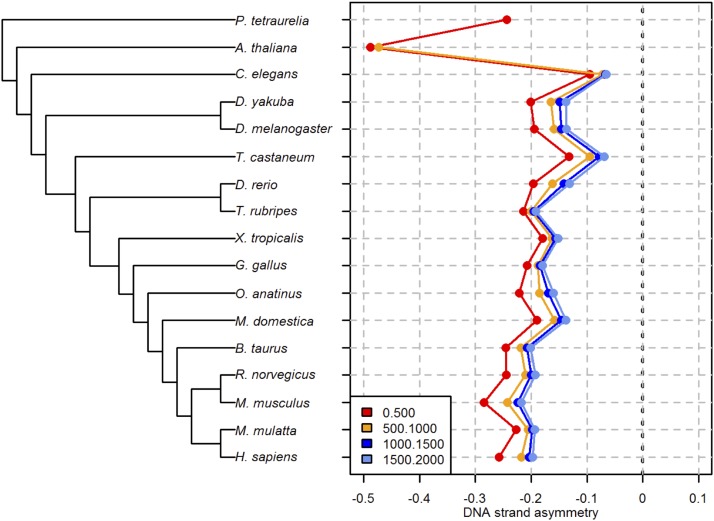
DNA strand asymmetry of the polyadenylation AATAAA motif (DSA_AATAAA_) in 500-nt intronic segments at increasing distances from the upstream 5′ss. Left panel: approximate phylogenetic relationships spanning ∼2 billion yr between the single-celled ciliate *P. tetraurelia* and *H. sapiens*. Right panel: DNA strand asymmetry of the AATAAA motif in four nonoverlapping intronic intervals. Only one interval (0–500 nt) was examined for *P*. *tetraurelia*, which has extremely short introns (25 bp on average; Aury *et al.* 2006). DSA_AATAAA_ was computed for samples that consist of ≥500 sequences.

Our observations do not meet this expectation for human, mouse, or *D*. *melanogaster* nor for any other of the species surveyed. We failed to detect DSA_AATAAA_ of ∼0 in the intronic interval immediately downstream of the 5′ss (Figure 2). Moreover, and much to our surprise, the most pronounced levels of selection against the AATAAA motif were detected for the intronic segment that is adjacent to the 5′ss, and, thus, within—rather than outside, as expected—the action range of telescripting. This pattern holds robustly for all of the species under examination (Figure 2 and Table S4 in File S1). Additionally, it is reinforced by the observation that, in *D*. *melanogaster*, a species with short and large introns, DSA_AATAAA_ is more negative in short introns than it is in larger introns (data not shown). We conclude that the vicinity of 5′ss-bound U1 does not reduce the selective pressure against cryptic intronic AATAAA PASs. Rather, it increases it.

### CPF access to introns could antagonize U1 recruitment at 5′ss

The widespread counter-selection of intronic AATAAA PASs that is illustrated in Figure 1 indicates that premature cleavage and polyadenylation is generally deleterious in eukaryotic cells. A nonmutually exclusive explanation for our observations is that CPF access to introns may have widespread negative effects on splicing. The pronounced selection pressure against intronic AATAAA PASs that are within the action range of telescripting (Figure 2) supports this latter hypothesis, and raises the possibility that CPF access to introns perturbs U1 recruitment to the upstream 5′ss. This idea is compatible with recent findings and theoretical models (Catania and Lynch 2008, 2013; Martinson 2011; Evsyukova *et al.* 2013; Lepennetier and Catania 2016).

If intronic AATAAA PASs truly disfavor U1 recruitment at 5′ss, then PASs that are in the vicinity of 5′ss may be intensely selected against. To test this hypothesis, we examined the DSA_AATAAA_ in nonoverlapping 100-nt intronic intervals that are at increasing distance from low- and high-scoring upstream 5′ss. We focused on human, mouse, and *D*. *melanogaster*, *i.e.*, the species for which telescripting has been described, and for which a model scoring the strength of 5′ and 3′ splice site signals is available (Yeo and Burge 2004).

**Figure 3:**
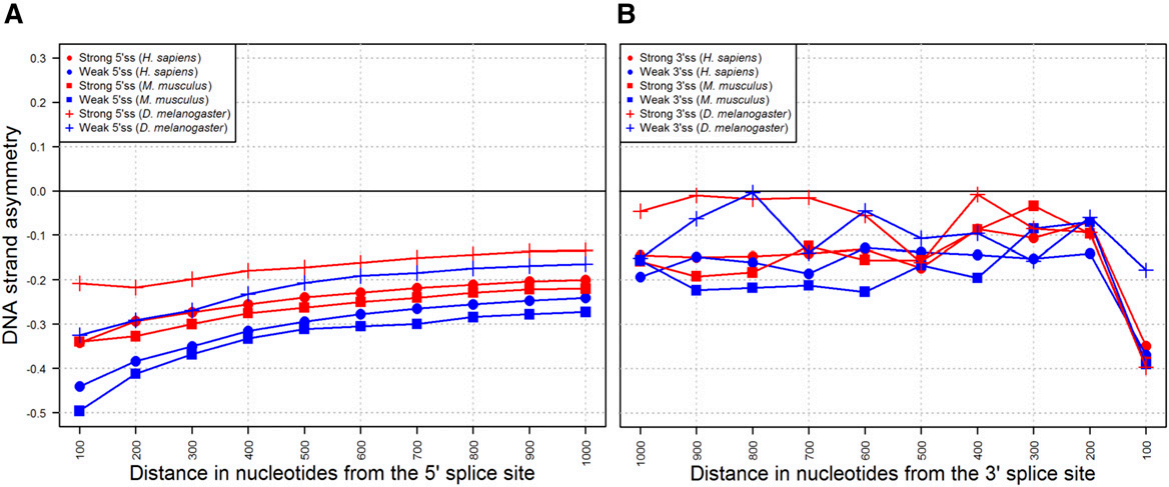
DNA strand asymmetry of the polyadenylation AATAAA motif (DSA_AATAAA_) in 100-nt intronic segments at increasing distances from low- and high-scoring upstream 5′ss (A) and downstream 3′ss (B). DSA_AATAAA_ was computed for samples that consist of ≥500 sequences. Low and high 5′ss (3′ss) scores are the lower and the upper quartile, respectively, of the 5′ss (3′ss) strength distribution generated for each of the species surveyed (human, mouse, and *D*. *melanogaster*).

In accord with our hypothesis, we found that the DSA_AATAAA_ becomes increasingly more negative as the distance between the intronic interval and the upstream 5′ss decreases. Moreover, we computed relatively more negative DSA_AATAAA_ scores when the upstream 5′ss is weak—a condition that presumably delays U1 recruitment (Figure 3A). In contrast, we detected no patterned variations when we studied intronic intervals that are at increasing distance from low- and high-scoring downstream 3′ss (Figure 3B). These observations lend support to the hypothesis that intron-bound CPFs may perturb the recruitment of U1 locally.

### DSA_AATAAA_ in genes with different functions

The results described above hint at a model where the strength of the 5′ss and the DSA of proximal AATAAA coexist in a dynamic equilibrium. We decided to explore the extent to which this equilibrium varies across genes with different functions and/or spatial-temporal patterns of expression. We employed *Drosophila* as a case study.

We leveraged the FlyBase database (http://flybase.org/static_pages/FBgg/browse.html) to extract randomly selected predefined sets of genes that encode for amine receptors, neuropeptides and hormone peptides, and spliceosomal components. We found that the DSA of AATAAA is indistinguishable from the DSA of its two anagrams TAAAAA and AAAAAT, employed as control motifs (*P* > 0.05) (Table S5 in File S1). Next, we examined genes activated by heat shock (Gonsalves *et al.* 2011). In the 1278 introns surveyed, we found that the DSA_AATAAA_ is rather negative, but nevertheless marginally within the range of the control motifs (AATAAA *vs.* TAAAAA: *P* = 0.08; AATAAA *vs.* AAAAAT: *P* = 0.04) (Table S5 in File S1). Finally, we used Guilgur *et al.* (2014) approach and extracted genes encoding early zygotic and maternally deposited transcripts from FlyBase (http://flybase.org/static_pages/rna-seq/rna-seq_profile_search.html). We found that the DSA_AATAAA_ is marginally comparable to that of control motifs for 585 early zygotic introns (AATAAA *vs.* TAAAAA: *P* = 0.07; AATAAA *vs.* AAAAAT: *P* = 0.03). However, it is significantly more negative for 5399 maternal introns (*P* < 0.0001) (Table S5 in File S1).

### DSA_AATAAA_ in highly and weakly expressed genes

**Figure 4:**
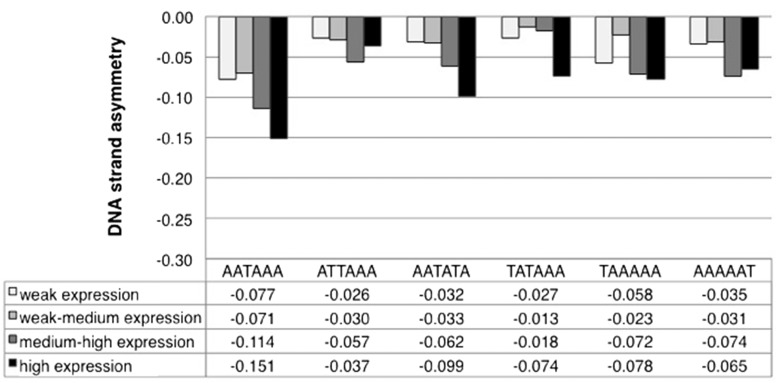
DNA strand asymmetry of intronic PAS(-like) six-mers and two AATAAA anagrams (TAAAAA and AAAAAT) in *D*. *melanogaster* genes with different expression levels. The gene expression values fall in the upper, lower, and intermediate (low-medium, and medium-high) quartiles of a distribution generated using ftp://flybase.org/flybase/associated_files/Gelbart.2010.10.13.tar.gz.

The nonsignificant asymmetry of the AATAAA PAS within the discussed intron sets could result in theory from weak purifying selection. In these circumstances, we might expect that genes with nonsignificant levels of intronic AATAAA asymmetry possess suboptimal splice sites. When we studied the 5′ ss strength, we found that all of the surveyed gene sets have larger-than-average 5′ splice site strength (Table S5 in File S1). We decided to explore this rationale further. Weakly expressed genes evolve under low levels of evolutionary constraints across several eukaryotes (Subramanian and Kumar 2004; Gout *et al.* 2010; Nabholz *et al.* 2013). Consequently, compared to highly expressed genes, weakly expressed genes may contain a less pronounced negative DSA_AATAAA_ and weaker 5′ splice sites. We tested this expectation using *D*. *melanogaster*. We found that the negative asymmetry of intronic AATAAA indeed tends to become more pronounced as the levels of gene expression increase (Figure 4). In weakly expressed genes, the degree of DNA strand asymmetry for AATAAA differs significantly from the control motif AAAAAT (*P* < 0.001), but not from TAAAAA (*P* = 0.12). In highly expressed genes, on the other hand, DSA_AATAAA_ is significantly more pronounced than the DSA of both control motifs (*P* < 0.0001). Additionally, as the levels of gene expression increase, also the 5′ and 3′ splice site strength increases (5′ss_low expression_ = 8.92, 5′ss_high expression_ = 9.16, Wilcoxon test *P* < 0.0001; 3′ss_low expression_ = 9.30, 3′ss_high expression_ = 9.75, Wilcoxon test *P* < 0.0001). To our knowledge, this is the first time that a relationship between splice site strength and levels of gene expression is documented for *Drosophila*. These observations align with the expectation that, under a weak selective regime, intronic AATAAA may tend to accumulate while U1s binding to 5′ss is suboptimal.

### CPF access to first and last introns may not be equally likely

The observations described above imply that CPFs may normally attempt to access intronic PASs along nascent transcripts, and that natural selection generally operates to counter this access. By continuing to study the DSA_AATAAA_, we may gain further insights into CPF-coupled dynamics. Namely, our analytical approach can provide information on how likely is CPF access to take place, and/or to be tolerated in some pre-mRNA regions more than in others.

In addressing this question, we computed the DSA_AATAAA_ for introns grouped according to their position along genes (first, internal, and last). We expected to detect no between-group variation in DSA_AATAAA_ scores if CPF access to introns is equally detrimental, and/or equally likely to occur in internal or peripheral pre-mRNA regions.

**Figure 5:**
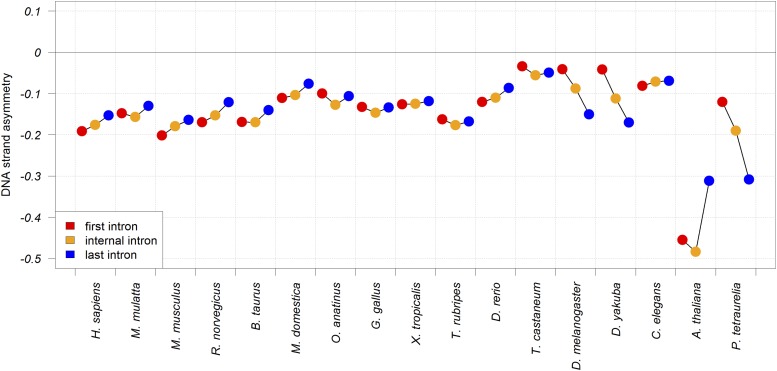
DNA strand asymmetry of the polyadenylation AATAAA motif (DSA_AATAAA_) in the first, internal, and last introns of the genes of 17 eukaryotic species.

Our study uncovered patterned variations. For many species surveyed (eight out of 17), the DSA_AATAAA_ is most negative in first introns, and least negative in last introns (*P* < 0.0001) (Figure 5 and Table S6 in File S1). On the other hand, in three species—*D*. *melanogaster*, *D*. *yakuba*, and *P*. *tetraurelia*—last introns show a lower DSA_AATAAA_ compared to first introns (*P* < 0.0001). Intriguingly, *Drosophila* and *Paramecium* are also the only organisms in our set for which we detected positive DSA_AATAAA_ in first exons (Table S3 in File S1). These variations are suggestive of differences in the intracellular environments of the eukaryotic organisms surveyed.

## Discussion

Studies that combine intracellular and selective processes may provide novel insights into the properties of modern genes. Telescripting, *i.e.*, the U1-dependent suppression of 5′ss-proximal polyadenylation signals (Gunderson *et al.* 1994), has emerged as a powerful mechanism to protect nascent eukaryotic transcripts from premature cleavage and polyadenylation (Kaida *et al.* 2010; Berg *et al.* 2012). Recently, we proposed that telescripting may impose constraints on exon–intron structure in *Drosophila* (Lepennetier and Catania 2016). Here, we asked whether CPFs, which during transcription are counteracted by U1 (Langemeier *et al.* 2013), also actively antagonize U1 recruitment. We attempted to gain insights into this question on a genomic scale.

We found that (1) cryptic PASs are universally selected against within eukaryotic introns, and (2) the levels of this counter-selection may vary both between species and along genes. Furthermore, (3) cryptic intronic PASs are intensely counter-selected in the vicinity of 5′ss (*i.e.*, within the action range of telescripting), more so if these 5′ss are weak. Finally, (4) the strength of 5′ss and the level of selection against cryptic intronic PASs may undergo compensatory changes. These observations open the possibility that U1 and CPFs compete for access to neighboring target sites (*i.e.*, 5′ss and 5′ss-proximal intronic PAS). This idea is central to U1-dependent definition—a model wherein the mechanisms of splice site recognition, gene structure evolution, and transcription activity are codependent (Catania and Lynch 2013).

Our study seems to capture dynamics that integrate transcription-coupled events with the action of natural selection. By considering multiple species simultaneously, our investigation provides insights into the generalizability of previous findings (Evsyukova *et al.* 2013; Lepennetier and Catania 2016). It also uncovers peculiarities within the eukaryotic tree of life. For example, we found that the DSA_AATAAA_ in the 3′ ends of *A. thaliana* and *T*. *castaneum* genes is close to zero (rather than being highly positive as it is in the remaining species). This hints at the possibility that in *A*. *thaliana* and *T*. *castaenum* the AATAAA PAS might not function as it does in many other eukaryotes. With regard to *A*. *thaliana*, our findings are consistent with a previous study where AATAAA, while being the most frequent six-mer in last exons’ terminal region, was detected within the predicted location in only ∼10% of 3′ UTRs (Loke *et al.* 2005). Although it is likely that AATAAA serves as a PAS motif in *A. thaliana*, it may be that this sequence motif may not wholly account for accurate cleavage and polyadenylation. Secondary structures at the 3′ UTR could influence the efficiency of AATAAA and other frequent PAS-like signals (Loke *et al.* 2005), in line with observations in mouse (Phillips *et al.* 1999). With respect to *T. castaneum*, the limited over-representation of AATAAA in this species’ genes tail seems to suggest that this six-mer might not operate as PAS in this species. Our additional observation that DSA_AATAAA_ in introns does not deviate from expectations is consistent with this idea. Further analyses are necessary to confirm this conclusion.

In another example of peculiarities uncovered by our study, *Drosophila* and *Paramecium* uniquely show positive DSA_AATAAA_ in their genes’ first exon and unusually relaxed selection against the AATAAA motif in their genes’ first intron. The causes of these between-species differences are still unspecified. By drawing from our previous suggestions (Catania and Lynch 2013; Lepennetier and Catania 2016), we propose a model where first-exon cryptic PASs—which the cap-proximal 5′ss can silence in *Drosophila* (Guo *et al.* 2011; Andersen *et al.* 2012)—advantageously divert CPFs, thus effectively aiding the splicing of the adjacent intron. Under these circumstances, cryptic PASs in the first introns of *Drosophila* and *Paramecium* genes would be, as is observed, under a more relaxed selective pressure compared to other species with no positive DSA_AATAAA_ in their genes’ first exons. These hypothetical dynamics need not only unfold at the pre-mRNA 5′ end. Moreover, they would be influenced by interdependent factors such as the efficiency of U1 recruitment to the 5′ss, intron size, and the local variations in the molar ratio between SFs and CPFs. In one example, a more elevated molar ratio of CPFs to SFs at the pre-mRNA 3′ end of *Drosophila* and *Paramecium* could potentially explain the more pronounced counter-selection of AATAAA PASs in the last intron of these species’ genes compared to the remaining species surveyed.

To conclude, our study leverages a simple computational approach to inferring transcription-coupled dynamics that are widespread across eukaryotes. It supplies new hypotheses for experimental research that may be tested in different organisms and through different approaches. If further substantiated, the notion that CPFs may critically impact the splicing process should have important consequences for the design of therapeutic approaches against splicing-associated human diseases and for the construction of models for the evolution of gene architecture and expression.

## Supplementary Material

Supplemental material is available online at www.g3journal.org/lookup/suppl/doi:10.1534/g3.117.041483/-/DC1.
