# Supplementary material for: Exploring the Impact of Cleavage and Polyadenylation Factors on Pre-mRNA Splicing Across Eukaryotes
Source: G3 (Bethesda). 2017 May 8;7(7):2107–14. doi: 10.1534/g3.117.041483 (PMC5499120; doi:10.1534/g3.117.041483)
Supplement: Supplementary file 1 [file 2107FileS1.docx]

**Supplemental Information**

**Exploring the impact of cleavage and polyadenylation factors on pre-mRNA splicing across eukaryotes**

Gildas Lepennetier and Francesco Catania

Institute for Evolution and Biodiversity, University of Münster, Hüfferstraße 1, 48149 Münster, Germany.

**Table S1.** Species surveyed and links for data retrieval.

| **Species** | **Links to genome DNA sequence and annotation file** |
| --- | --- |
| *Homo sapiens* | <ftp://ftp.ensembl.org/pub/release-84/fasta/homo_sapiens/dna/Homo_sapiens.GRCh38.dna_sm.primary_assembly.fa.gz>  <ftp://ftp.ensembl.org/pub/release-84/gtf/homo_sapiens/Homo_sapiens.GRCh38.84.gtf.gz> |
| *Macaca mulatta* | <ftp://ftp.ensembl.org/pub/release-84/fasta/macaca_mulatta/dna/Macaca_mulatta.MMUL_1.dna_sm.toplevel.fa.gz>  <ftp://ftp.ensembl.org/pub/release-84/gtf/macaca_mulatta/Macaca_mulatta.MMUL_1.84.gtf.gz> |
| *Mus musculus* | <ftp://ftp.ensembl.org/pub/release-84/fasta/mus_musculus/dna/Mus_musculus.GRCm38.dna_sm.toplevel.fa.gz>  <ftp://ftp.ensembl.org/pub/release-84/gtf/mus_musculus/Mus_musculus.GRCm38.84.gtf.gz> |
| *Rattus norvegicus* | <ftp://ftp.ensembl.org/pub/release-84/fasta/rattus_norvegicus/dna/Rattus_norvegicus.Rnor_6.0.dna_sm.toplevel.fa.gz>  <ftp://ftp.ensembl.org/pub/release-84/gtf/rattus_norvegicus/Rattus_norvegicus.Rnor_6.0.84.gtf.gz> |
| *Drosophila melanogaster* | <ftp://ftp.ensembl.org/pub/release-84/fasta/drosophila_melanogaster/dna/Drosophila_melanogaster.BDGP6.dna_sm.toplevel.fa.gz>  <ftp://ftp.ensembl.org/pub/release-84/gtf/drosophila_melanogaster/Drosophila_melanogaster.BDGP6.84.gtf.gz> |
| *Tribolium castaneum* | <ftp://ftp.ensemblgenomes.org/pub/metazoa/release-31/fasta/tribolium_castaneum/dna/Tribolium_castaneum.Tcas3.31.dna_rm.toplevel.fa.gz>  <ftp://ftp.ensemblgenomes.org/pub/metazoa/release-31/gff3/tribolium_castaneum/Tribolium_castaneum.Tcas3.31.gff3.gz> |
| *Bos taurus* | <ftp://ftp.ensembl.org/pub/release-84/fasta/bos_taurus/dna/Bos_taurus.UMD3.1.dna_sm.toplevel.fa.gz>  <ftp://ftp.ensembl.org/pub/release-84/gtf/bos_taurus/Bos_taurus.UMD3.1.84.gtf.gz> |
| *Gallus gallus* | <ftp://ftp.ensembl.org/pub/release-84/fasta/gallus_gallus/dna/Gallus_gallus.Galgal4.dna_sm.toplevel.fa.gz>  <ftp://ftp.ensembl.org/pub/release-84/gtf/gallus_gallus/Gallus_gallus.Galgal4.84.gtf.gz> |
| *Ornithorhynchus anatinus* | <ftp://ftp.ensembl.org/pub/release-84/fasta/ornithorhynchus_anatinus/dna/Ornithorhynchus_anatinus.OANA5.dna_sm.toplevel.fa.gz>  <ftp://ftp.ensembl.org/pub/release-84/gtf/ornithorhynchus_anatinus/Ornithorhynchus_anatinus.OANA5.84.gtf.gz> |
| *Xenopus tropicalis* | <ftp://ftp.ensembl.org/pub/release-84/fasta/xenopus_tropicalis/dna/Xenopus_tropicalis.JGI_4.2.dna_sm.toplevel.fa.gz>  <ftp://ftp.ensembl.org/pub/release-84/gtf/xenopus_tropicalis/Xenopus_tropicalis.JGI_4.2.84.gtf.gz> |
| *Monodelphis domestica* | <ftp://ftp.ensembl.org/pub/release-84/fasta/monodelphis_domestica/dna/Monodelphis_domestica.BROADO5.dna_sm.toplevel.fa.gz>  <ftp://ftp.ensembl.org/pub/release-84/gtf/monodelphis_domestica/Monodelphis_domestica.BROADO5.84.gtf.gz> |
| *Takifugu rubripes* | <ftp://ftp.ensembl.org/pub/release-84/fasta/takifugu_rubripes/dna/Takifugu_rubripes.FUGU4.dna_sm.toplevel.fa.gz>  <ftp://ftp.ensembl.org/pub/release-84/gtf/takifugu_rubripes/Takifugu_rubripes.FUGU4.84.gtf.gz> |
| *Danio rerio* | <ftp://ftp.ensembl.org/pub/release-84/fasta/danio_rerio/dna/Danio_rerio.GRCz10.dna_sm.toplevel.fa.gz>  <ftp://ftp.ensembl.org/pub/release-84/gtf/danio_rerio/Danio_rerio.GRCz10.84.gtf.gz> |
| *Caenorhabditis elegans* | <ftp://ftp.ensembl.org/pub/release-84/fasta/caenorhabditis_elegans/dna/Caenorhabditis_elegans.WBcel235.dna.toplevel.fa.gz>  <ftp://ftp.ensembl.org/pub/release-84/gtf/caenorhabditis_elegans/Caenorhabditis_elegans.WBcel235.84.gtf.gz> |
| *Drosophila yakuba* | <ftp://ftp.flybase.net/genomes/Drosophila_yakuba/dyak_r1.04_FB2015_01/fasta/dyak-all-chromosome-r1.04.fasta.gz>  <ftp://ftp.flybase.net/genomes/Drosophila_yakuba/dyak_r1.04_FB2015_01/gtf/dyak-all-r1.04.gtf.gz> |
| *Arabidopsis thaliana* | <ftp://ftp.arabidopsis.org/home/tair/Genes/TAIR10_genome_release/TAIR10_chromosome_files/TAIR10_chr_all.fas>  <ftp://ftp.arabidopsis.org/home/tair/Genes/TAIR10_genome_release/TAIR10_gff3/TAIR10_GFF3_genes.gff> |
| *Paramecium tetraurelia* | <http://paramecium.cgm.cnrs-gif.fr/download/fasta/assemblies/ptetraurelia_mac_51.fa>  <http://paramecium.cgm.cnrs-gif.fr/download/gff/ptetraurelia_CDS_v1_pt_51.gff3.gz> |

**Table S2.** DNA strand asymmetry (DSA) analysis on all possible 4096 6-mers, selected results. TAAAAA and AAAAAT are included as control motifs to estimate the significance of the AATAAA’s relative deficit within the DNA sense strand. Only motifs with a minimum of 100 occurrences in the DNA antisense strand are considered for assessing the rank of DSA_AATAAA_.

| **Species** | **6-mer with most negative [DSA value]** | **DSA_AATAAA_ rank in a sorted list of DSA estimates for 4096 6-mers** | **DSA_AATAAA_** | **DSA_TAAAAA_** | **DSA_AAAAAT_** | **AT% in introns** | **DSA_AATAAA_**  ***<* DSA_TAAAAA_** | **DSA_AATAAA_**  ***<* DSA_AAAAAT_** |
| --- | --- | --- | --- | --- | --- | --- | --- | --- |
| *H. sapiens* | CAACGA [-0.2611] | 109 (3%) | -0.1775 | -0.1454 | -0.1308 | 59 | ***** | ***** |
| *M. mulatta* | CAACGA [-0.2735] | 131 (3%) | -0.1508 | -0.1228 | -0.1055 | 59 | ***** | ***** |
| *M. musculus* | CAACAC [-0.2301] | 60 (1%) | -0.1832 | -0.1432 | -0.1389 | 57 | ***** | ***** |
| *R. norvegicus* | ACGCAA [-0.2395] | 78 (2%) | -0.1541 | -0.1207 | -0.1051 | 57 | ***** | ***** |
| *B. taurus* | CAACGA [-0.3185] | 158 (4%) | -0.1670 | -0.1282 | -0.1108 | 58 | ***** | ***** |
| *M. domestica* | ACAACG [-0.1647] | 140 (3%) | -0.1012 | -0.0938 | -0.0860 | 62 | ***** | ***** |
| *O. anatinus* | TACGAC [-0.2862] | 554 (14%) | -0.1199 | -0.1091 | -0.0944 | 57 | **** | ***** |
| *G. gallus* | ACACAC [-0.3897] | 656 (16%) | -0.1424 | -0.1210 | -0.1020 | 59 | ***** | ***** |
| *X. tropicalis* | ACACAA [-0.1334] | 150 (4%) | -0.1238 | -0.1017 | -0.0879 | 61 | ***** | ***** |
| *T. rubripes* | AACAAC [-0.185] | 32 (1%) | -0.1726 | -0.1542 | -0.1440 | 56 | ***** | ***** |
| *D. rerio* | GACAGA [-0.1095] | 21 (1%) | -0.1100 | -0.0821 | -0.0708 | 64 | ***** | ***** |
| *T. castaneum* | CATGCA [-0.0645] | 231 (6%) | -0.0445 | -0.0469 | -0.0484 | 67 | *P = 0.27* | *P = 0.07* |
| *D. melanogaster* | GGTAAG [-0.1972] | 170 (4%) | -0.0777 | -0.0388 | -0.0371 | 60 | ***** | ***** |
| *D. yakuba* | GGTAAG [-0.2572] | 264 (6%) | -0.0893 | -0.0490 | -0.0416 | 60 | ***** | ***** |
| *C. elegans* | GGTAAG [-0.2015] | 465 (11%) | -0.0742 | 0.0054 | 0.0087 | 68 | ***** | ***** |
| *A. thaliana* | ACAGAG [-0.6235] | 255 (6%) | -0.4441 | -0.3361 | -0.3834 | 68 | ***** | ***** |
| *P. tetraurelia*^‡^ | CTAGGG [-0.9661] | 1002 (24%) | -0.2121 | -0.1879 | -0.2264 | 78 | *** | *P = 0.08* |

*** = *P* < 0.01; **** = *P* < 0.001; ***** = *P* < 0.0001. A chi-square test is performed to determine whether the observed counts of AATAAA within sense and antisense DNA strand are significantly different from the expected counts that are based on the occurrences of the control motif TAAAAA or AAAAAT.

^‡^ = Untrimmed introns are used for this species.

**Table S3.** DNA strand asymmetry of the AATAAA motif and median (average) size of first, internal and last exon. In the case of last exons, DSA values are provided for the whole exon and for the last 100 nucleotides separately.

| **Species** | **Intragenic position** | **Observations** | **DNA strand asymmetry (whole exon)** | **Median (average) size in base pairs** | **DNA strand asymmetry (last 100 nucleotides)** |
| --- | --- | --- | --- | --- | --- |
| *H. sapiens* | first | 12,276 | -0.166 | 159 (248) |  |
|  | internal | 60,200 | 0.135 | 123 (151) |  |
|  | last | 12,276 | 0.031 | 394 (951) | 0.597 |
| *M. mulatta* | first | 15,400 | -0.002 | 139 (218) |  |
|  | internal | 111,174 | 0.111 | 118 (143) |  |
|  | last | 15,400 | 0.089 | 308 (583) | 0.570 |
| *M. musculus* | first | 13,956 | -0.118 | 165 (240) |  |
|  | internal | 79,292 | 0.112 | 124 (152) |  |
|  | last | 13,956 | 0.084 | 587 (1044) | 0.633 |
| *R. norvegicus* | first | 15,913 | -0.073 | 166 (239) |  |
|  | internal | 131,985 | 0.125 | 121 (147) |  |
|  | last | 15,913 | 0.149 | 537 (886) | 0.611 |
| *B. taurus* | first | 15,789 | -0.013 | 138 (210) |  |
|  | internal | 135,306 | 0.119 | 121 (144) |  |
|  | last | 15,789 | 0.164 | 408 (624) | 0.601 |
| *M. domestica* | first | 14,014 | -0.046 | 238 (359) |  |
|  | internal | 122,607 | 0.095 | 123 (150) |  |
|  | last | 14,014 | 0.028 | 643 (1324) | 0.378 |
| *O. anatinus* | first | 2,285 | -0.080 | 159 (232) |  |
|  | internal | 20,096 | 0.090 | 118 (139) |  |
|  | last | 2,285 | 0.046 | 331 (950) | 0.416 |
| *G. gallus* | first | 4,780 | -0.003 | 139 (225) |  |
|  | internal | 48,809 | 0.164 | 123 (145) |  |
|  | last | 4,781 | 0.068 | 561 (960) | 0.576 |
| *X. tropicalis* | first | 15,250 | -0.120 | 125 (191) |  |
|  | internal | 135,662 | 0.073 | 117 (137) |  |
|  | last | 15,250 | 0.007 | 231 (502) | 0.449 |
| *T. rubripes* | first | 16,439 | -0.098 | 114 (164) |  |
|  | internal | 138,308 | 0.046 | 120 (137) |  |
|  | last | 16,439 | 0.139 | 129 (182) | 0.313 |
| *D. rerio* | first | 18,402 | -0.182 | 177 (246) |  |
|  | internal | 130,427 | 0.180 | 123 (153) |  |
|  | last | 18,402 | -0.039 | 495 (818) | 0.481 |
| *T. castaneum* | first | 9,794 | -0.068 | 114 (202) |  |
|  | internal | 30,543 | -0.073 | 200 (294) |  |
|  | last | 9,794 | 0.018 | 209 (337) | 0.045 |
| *D. melanogaster* | first | 7,253 | 0.256 | 230 (340) |  |
|  | internal | 19,777 | 0.142 | 218 (390) |  |
|  | last | 7,253 | 0.225 | 596 (849) | 0.553 |
| *D. yakuba* | first | 7,393 | 0.262 | 237 (351) |  |
|  | internal | 19,436 | 0.117 | 216 (382) |  |
|  | last | 7,393 | 0.204 | 516 (741) | 0.510 |
| *C. elegans* | first | 16,677 | -0.406 | 126 (157) |  |
|  | internal | 59,899 | -0.203 | 152 (215) |  |
|  | last | 16,677 | -0.086 | 254 (303) | 0.152 |
| *A. thaliana* | first | 11,538 | -0.081 | 247 (316) |  |
|  | internal | 54,882 | -0.064 | 116 (171) |  |
|  | last | 11,534 | -0.087 | 382 (451) | -0.045 |
| *P. tetraurelia* | first | 22,643 | 0.387 | 175 (294) |  |
|  | internal | 35,317 | 0.348 | 220 (399) |  |
|  | last | 22,643 | 0.414 | 198 (350) | 0.464 |

**Table S4.** Counts of AATAAA in sense and antisense DNA strand in intronic regions that are within 500 base pairs (bp) or between 500 bp and 1000 bp away from the upstream 5'ss. A chi-square test is used to test for an association between AATAAA counts and distance from the upstream 5'ss (*** = *P* < 0.0001).

| **Species** | **Number of AATAAA occurrences** | | | | ***P-value*** |
| --- | --- | --- | --- | --- | --- |
|  | (**A**) <500 bp from 5'ss | | (**B**) ≥500 - <1000 bp from 5'ss | |  |
|  | (**A1**) *DNA sense strand* | (**A2**) *DNA antisense strand* | (**B1**) *DNA sense strand* | (**B2**) *DNA antisense strand* |  |
| *H. sapiens* | 24817 | 41865 | 24028 | 33979 | A1 < B1 ***** |
| *M. mulatta* | 28300 | 44727 | 21289 | 30394 | A1 < B1 ***** |
| *M. musculus* | 24762 | 44305 | 24649 | 36717 | A1 < B1 ***** |
| *R. norvegicus* | 25588 | 41986 | 21918 | 30678 | A1 < B1 ***** |
| *B. taurus* | 32366 | 53186 | 24967 | 36001 | A1 < B1 ***** |
| *M. domestica* | 44109 | 64402 | 36441 | 46187 | A1 < B1 ***** |
| *O. anatinus* | 4483 | 6998 | 3322 | 4327 | A1 < B1 ***** |
| *G. gallus* | 12281 | 18605 | 8339 | 11466 | A1 < B1 ***** |
| *X. tropicalis* | 50188 | 71802 | 29278 | 38256 | A1 < B1 ***** |
| *T. rubripes* | 29157 | 44561 | 10044 | 13779 | A1 < B1 ***** |
| *D. rerio* | 56828 | 84197 | 33289 | 40510 | A1 < B1 ***** |
| *T. castaneum* | 18727 | 24170 | 12688 | 13663 | A1 < B1 ***** |
| *D. melanogaster* | 7516 | 11066 | 3807 | 4482 | A1 < B1 ***** |
| *D. yakuba* | 7493 | 11176 | 3568 | 4172 | A1 < B1 ***** |
| *C. elegans* | 26352 | 32555 | 6999 | 6640 | A1 < B1 ***** |
| *A. thaliana* | 7740 | 21942 | 750 | 1278 | A1 < B1 ***** |
| *P. tetraurelia* | 5678 | 8735 | - | - | - |

**Table S5.** DNA strand asymmetry of PAS(-like) 6-mers, two AATAAA anagrams, and splice site strength for sets of genes with different functions and/or spatial-temporal patterns of expression. The average splice site strength for the introns in each gene subset was compared to the overall average splice strength in Drosophila. The significance of the deviation (ns = not significant; * = *P* < 0.01; ** = *P* < 0.001; *** = *P* < 0.0001) was calculated comparing the frequency with which the average 5'ss and 3'ss strength for the relevant gene set was higher or lower than the counterpart measured for same-sized random sample of genes (1,000,000 samplings with replacement).

|  |  | **No of introns surveyed** | **Occurrences in DNA sense strand** | **Occurrences in DNA antisense strand** | **DSA** | **Average 5' splice site strength** | **Average 3' splice site strength** |
| --- | --- | --- | --- | --- | --- | --- | --- |
|  |  |  |  |  |  |  |  |
| **Neuropeptides/hormone peptides** |  | 77 |  |  |  | 9.792*** | 9.818^ns^ |
|  | AATAAA |  | 178 | 167 | 0.0319 |  |  |
|  | ATTAAA |  | 172 | 186 | -0.0391 |  |  |
|  | AATATA |  | 116 | 107 | 0.0404 |  |  |
|  | TATAAA |  | 131 | 111 | 0.0826 |  |  |
|  | TAAAAA |  | 155 | 171 | -0.0491 |  |  |
|  | AAAAAT |  | 197 | 199 | -0.0051 |  |  |
|  |  |  |  |  |  |  |  |
| **Amine receptors** |  | 85 |  |  |  | 10.052*** | 9.993^ns^ |
|  | AATAAA |  | 509 | 633 | -0.1086 |  |  |
|  | ATTAAA |  | 509 | 503 | 0.0059 |  |  |
|  | AATATA |  | 314 | 306 | 0.0129 |  |  |
|  | TATAAA |  | 301 | 308 | -0.0114 |  |  |
|  | TAAAAA |  | 442 | 471 | -0.0318 |  |  |
|  | AAAAAT |  | 561 | 632 | -0.0595 |  |  |
|  |  |  |  |  |  |  |  |
| **Spliceosomal proteins** |  | 100 |  |  |  | 9.561* | 10.733*** |
|  | AATAAA |  | 8 | 13 | -0.2381 |  |  |
|  | ATTAAA |  | 12 | 12 | 0 |  |  |
|  | AATATA |  | 6 | 16 | -0.4545 |  |  |
|  | TATAAA |  | 8 | 16 | -0.3333 |  |  |
|  | TAAAAA |  | 10 | 15 | -0.2000 |  |  |
|  | AAAAAT |  | 9 | 19 | -0.3571 |  |  |
|  |  |  |  |  |  |  |  |
| **Heat shock activated genes** |  | 1278 |  |  |  | 9.378*** | 9.830** |
|  | AATAAA |  | 1737 | 2161 | -0.1088 |  |  |
|  | ATTAAA |  | 1485 | 1578 | -0.0304 |  |  |
|  | AATATA |  | 1151 | 1314 | -0.0661 |  |  |
|  | TATAAA |  | 1172 | 1235 | -0.0262 |  |  |
|  | TAAAAA |  | 1749 | 2005 | -0.0682 |  |  |
|  | AAAAAT |  | 2118 | 2403 | -0.0630 |  |  |
|  |  |  |  |  |  |  |  |
| **Early zygotic transcripts** |  | 585 |  |  |  | 9.994*** | 10.191*** |
|  | AATAAA |  | 1251 | 1473 | -0.0815 |  |  |
|  | ATTAAA |  | 1064 | 1107 | -0.0198 |  |  |
|  | AATATA |  | 740 | 817 | -0.0495 |  |  |
|  | TATAAA |  | 762 | 803 | -0.0262 |  |  |
|  | TAAAAA |  | 1225 | 1279 | -0.0216 |  |  |
|  | AAAAAT |  | 1568 | 1677 | -0.0336 |  |  |
|  |  |  |  |  |  |  |  |
| **Maternally deposited transcripts** |  | 5399 |  |  |  | 9.281*** | 9.587^ns^ |
|  | AATAAA |  | 2635 | 3738 | -0.1731 |  |  |
|  | ATTAAA |  | 2473 | 2773 | -0.0572 |  |  |
|  | AATATA |  | 2132 | 2564 | -0.0920 |  |  |
|  | TATAAA |  | 2114 | 2366 | -0.0563 |  |  |
|  | TAAAAA |  | 2954 | 3464 | -0.0795 |  |  |
|  | AAAAAT |  | 3478 | 3976 | -0.0668 |  |  |
|  |  |  |  |  |  |  |  |

**Table S6.** DNA strand asymmetry of the AATAAA PAS in first, internal, and last introns. The count of AATAAA motifs detected in the sense strand and in the antisense strand, the total number of introns screened, and the median (average) size of the introns sets per position are provided. A chi-square test is performed to test for an association between AATAAA counts and intron position (ns = not significant; * = *P* < 0.01; ** = *P* < 0.001; *** = *P* < 0.0001).

| **Species** | **Intragenic position** | **Observations** | **Median (average) size in base pairs** | **Sense strand (5' to 3')** | **Antisense strand (3' to 5')** | **DNA strand asymmetry** | ***P-value***  ***DSA_AATAAA_ First* vs. *DSA_AATAAA_ Last*** |
| --- | --- | --- | --- | --- | --- | --- | --- |
| *H. sapiens* | First | 12,276 | 2753 (11729) | 123121 | 181416 | -0.1914 | First < Last ***** |
|  | Internal | 60,200 | 1506 (5113) | 289390 | 413241 | -0.1763 |  |
|  | Last | 12,276 | 1469 (4928) | 55803 | 75941 | -0.1529 |  |
| *M. mulatta* | First | 15,400 | 2262 (10471) | 73518 | 99022 | -0.1478 | First < Last ***** |
|  | Internal | 111,174 | 1293 (4211) | 227068 | 311640 | -0.1570 |  |
|  | Last | 15,400 | 1107 (3814) | 27220 | 35335 | -0.1297 |  |
| *M. musculus* | First | 13,956 | 2317 (9865) | 95370 | 143530 | -0.2016 | First < Last ***** |
|  | Internal | 79,292 | 1275 (3908) | 231905 | 333018 | -0.1790 |  |
|  | Last | 13,956 | 1199 (3532) | 35143 | 48945 | -0.1641 |  |
| *R. norvegicus* | First | 15,913 | 2489 (9398) | 57672 | 81289 | -0.1700 | First < Last ***** |
|  | Internal | 131,985 | 1168 (3404) | 191244 | 260502 | -0.1533 |  |
|  | Last | 15,913 | 1084 (3138) | 20405 | 26041 | -0.1213 |  |
| *B. taurus* | First | 15,789 | 2227 (9399) | 61626 | 86739 | -0.1693 | First < Last ***** |
|  | Internal | 135,306 | 1118 (3413) | 213840 | 301245 | -0.1697 |  |
|  | Last | 15,789 | 999 (3115) | 22371 | 29663 | -0.1401 |  |
| *M. domestica* | First | 14,014 | 3758 (15268) | 134762 | 168441 | -0.1111 | First < Last ***** |
|  | Internal | 122,607 | 1691 (5511) | 464229 | 571460 | -0.1035 |  |
|  | Last | 14,014 | 1569 (5853) | 53819 | 62696 | -0.0762 |  |
| *O. anatinus* | First | 2,285 | 2591 (10424) | 8578 | 10480 | -0.0998 | First = Last ^ns^ |
|  | Internal | 20,096 | 1493 (3858) | 31309 | 40474 | -0.1277 |  |
|  | Last | 2,285 | 1565 (4624) | 3871 | 4790 | -0.1061 |  |
| *G. gallus* | First | 4,780 | 1143 (5285) | 47275 | 61752 | -0.1328 | First = Last ^ns^ |
|  | Internal | 48,809 | 650 (1886) | 187849 | 252319 | -0.1465 |  |
|  | Last | 4,781 | 669 (1780) | 18526 | 24237 | -0.1336 |  |
| *X. tropicalis* | First | 15,250 | 1572 (4601) | 44877 | 57842 | -0.1262 | First = Last ^ns^ |
|  | Internal | 135,662 | 786 (1852) | 174992 | 224812 | -0.1246 |  |
|  | Last | 15,250 | 865 (1848) | 19062 | 24183 | -0.1184 |  |
| *T. rubripes* | First | 16,439 | 357 (1104) | 12905 | 17925 | -0.1628 | First = Last ^ns^ |
|  | Internal | 138,308 | 133 (514) | 48749 | 69669 | -0.1767 |  |
|  | Last | 16,439 | 148 (450) | 5300 | 7432 | -0.1675 |  |
| *D. rerio* | First | 18,402 | 1728 (5951) | 77149 | 98236 | -0.1202 | First < Last ***** |
|  | Internal | 130,427 | 918 (2358) | 210843 | 263223 | -0.1105 |  |
|  | Last | 18,402 | 1517 (2702) | 32765 | 38955 | -0.0863 |  |
| *T. castaneum* | First | 9,794 | 60 (1564) | 28580 | 30586 | -0.0339 | First > Last *** |
|  | Internal | 30,543 | 54 (845) | 48578 | 54331 | -0.0559 |  |
|  | Last | 9,794 | 54 (949) | 16446 | 18165 | -0.0497 |  |
| *D. melanogaster* | First | 7,253 | 149 (1614) | 15632 | 16973 | -0.0411 | First > Last ***** |
|  | Internal | 19,777 | 69 (1090) | 27249 | 32495 | -0.0878 |  |
|  | Last | 7,253 | 65 (394) | 3364 | 4553 | -0.1502 |  |
| *D. yakuba* | First | 7,393 | 146 (1489) | 14327 | 15578 | -0.0418 | First > Last ***** |
|  | Internal | 19,436 | 68 (742) | 16745 | 20964 | -0.1119 |  |
|  | Last | 7,393 | 65 (361) | 2982 | 4207 | -0.1704 |  |
| *C. elegans* | First | 16,677 | 79 (350) | 8921 | 10506 | -0.0816 | First = Last ^ns^ |
|  | Internal | 59,899 | 58 (264) | 23273 | 26854 | -0.0714 |  |
|  | Last | 16,677 | 62 (256) | 6605 | 7590 | -0.0694 |  |
| *A. thaliana* | First | 11,538 | 154 (245) | 2853 | 7613 | -0.4548 | First < Last ***** |
|  | Internal | 54,882 | 97 (143) | 6949 | 19961 | -0.4835 |  |
|  | Last | 11,534 | 96 (153) | 2224 | 4236 | -0.3115 |  |
| *P. tetraurelia* | First | 22,643 | 25 (25) | 1432 | 1825 | -0.1207 | First > Last ***** |
|  | Internal | 35,317 | 25 (25) | 2272 | 3340 | -0.1903 |  |
|  | Last | 22,643 | 25 (25) | 1403 | 2654 | -0.3084 |  |
